# MtbHLH1, a bHLH transcription factor involved in *Medicago truncatula* nodule vascular patterning and nodule to plant metabolic exchanges

**DOI:** 10.1111/j.1469-8137.2011.03718.x

**Published:** 2011-07

**Authors:** Laurence Godiard, Agnès Lepage, Sandra Moreau, Damien Laporte, Marion Verdenaud, Ton Timmers, Pascal Gamas

**Affiliations:** 1 Laboratoire des Interactions Plantes Microorganismes, Unité Mixte de Recherche, Institut National de la Recherche Agronomique – Centre National de la Recherche Scientifique 441/2594F–31320 Castanet Tolosan, France; 2 Jian-Qiu Wu's laboratory, Ohio State University612 Biosciences Building, 484 W 12th Ave, Columbus, OH 43210, USA

**Keywords:** auxin regulation, bHLH, CRES-T, LOB, nodulation, transcription factor, uninfected cells, vascularization

## Abstract

- This study aimed at defining the role of a basic helix–loop–helix (bHLH) transcription factor gene from *Medicago truncatula, MtbHLH1,* whose expression is upregulated during the development of root nodules produced upon infection by rhizobia bacteria.
- We used *MtbHLH1* promoter::GUS fusions and quantitative reverse-transcription polymerase chain reaction analyses to finely characterize the *MtbHLH1* expression pattern*.* We altered MtbHLH1 function by expressing a dominantly repressed construct (CRES-T approach) and looked for possible MtbHLH1 target genes by transcriptomics*.*
- We found that *MtbHLH1* is expressed in nodule primordia cells derived from pericycle divisions, in nodule vascular bundles (VBs) and in uninfected cells of the nitrogen (N) fixation zone. *MtbHLH1* is also expressed in root tips, lateral root primordia cells and root VBs, and induced upon auxin treatment. Altering MtbHLH1 function led to an unusual phenotype, with a modified patterning of nodule VB development and a reduced growth of aerial parts of the plant, even though the nodules were able to fix atmospheric N. Several putative MtbHLH1 regulated genes were identified, including an asparagine synthase and a LOB (lateral organ boundary) transcription factor.
- Our results suggest that the *MtbHLH1* gene is involved in the control of nodule vasculature patterning and nutrient exchanges between nodules and roots.

## Introduction

Legumes play a crucial role in both ecological and agricultural systems by their capacity to establish a symbiosis with nitrogen-fixing bacteria called rhizobia. This process involves the formation of a specific organ, the root nodule and relies on the mutual recognition of both partners via molecular signals and activation of a plant symbiotic program.

The root nodule provides rhizobia with a carbon (C) source derived from photosynthesis and an appropriate cellular environment allowing the bacterial nitrogenase to fix atmospheric nitrogen (N). In temperate legumes, represented by the model legume *Medicago truncatula*, the nodule is a highly structured organ with an indeterminate growth, resulting from the activity of an apical meristematic region (also called zone I) and the differentiation of several peripheral and central tissues.

The peripheral tissues include the nodule cortex, the nodule endodermis and the nodule parenchyma in which the nodule vascular bundles (VBs) are located; in indeterminate nodules, the VBs are connected at their proximal end to the root vasculature and are open at the distal end. The nodule VBs are composed of xylem and phloem vessels in parenchyma cells surrounded by a pericycle cell layer and a vascular endodermis, which constitutes an apoplastic barrier between the VB and the nodule central tissues (Schubert, 2007).

The central nodule tissues comprise an infection zone II where *Sinorhizobium meliloti* bacteria are released from transcellular infection threads (ITs) and where coordinated differentiation of both plant and bacterial cells takes place, accompanied by several cycles of endoreduplication in infected plant cells. This leads to the formation of the fixation zone III, followed at its proximal part by the senescence zone IV where both symbionts degenerate (Vasse *et al.*, 1990). The fixation zone III occupies the largest region of mature nodules and is composed of two types of cells: large infected cells (ICs), in which N fixation is carried out by terminally differentiated bacteroids, and smaller uninfected cells (UCs), which are interspersed between the ICs and whose function is still unclear. The nodule is a root organ with metabolite fluxes playing an essential role both inward, with photosynthates brought by the phloem providing a C source and energy for the N fixation and assimilation processes, and outward, with the ensuing nitrogenous compounds which are transported by the xylem. In determinate nodules, UCs are specifically involved in synthesis and transport of ureides, the major fixed N product transported from these nodule types. In indeterminate nodules, no specific role has yet been assigned to UCs in the transport of asparagine, the main product of N fixation (for review, Vance, 2002). The universal presence of UCs in the infected tissue of determinate and indeterminate nodules with ITs suggests, however, that they have an important role in nodule functioning (Sprent & James, 2007).

In the past two decades, genetics and molecular approaches, using the model legumes *Lotus japonicus* and *M. truncatula*, have led to the identification of plant transcription factor (TF) genes involved in the initial symbiotic stages associated with Nod factor perception and signal transduction (Stougaard, 2000; Oldroyd & Downie, 2008; Libault *et al.*, 2009). However, very few regulatory host genes have been associated with the structural development of the functional nodule, and particularly VB development. One notable exception is the Krüppel-like zinc finger TF gene, *Mszpt2-1*, in *Medicago sativa,* which is strongly induced in the nodule VB upon *S. meliloti* infection. Plants expressing an antisense *Mszpt2-1* construct develop nonfixing nodules, in which bacterial invasion and differentiation of the central fixation zone is arrested (Frugier *et al.*, 2000). More recently class-III homeodomain-leucine zipper (*HD-ZIPIII*) genes have been described to be expressed in root and nodule vascular bundles as well as in the nodule zone I and II (Boualem *et al.*, 2008). Overexpressing MIR166, which targets these genes, reduced the number of nodules and lateral roots, and strongly modified the vascular bundle development in roots (Boualem *et al.*, 2008), but nodule vascularization was not examined.

Basic helix–loop–helix (bHLH) proteins constitute one of the largest TF families, widely distributed in all eukaryotes (Stevens *et al.*, 2008), and involved in a variety of signalling and developmental processes in plants (Heim *et al.*, 2003; Toledo-Ortiz *et al.*, 2003; Li *et al.*, 2006; Carretero-Paulet *et al.*, 2010). The bHLH signature motif is 60 amino acids long and composed of a basic region of 15–20 residues, followed by the HLH region composed of two amphipathic helices consisting of hydrophobic residues linked by a more divergent loop region. The HLH region is a protein–protein interaction domain and the basic regions of two homodimerized or heterodimerized bHLHs are able to bind DNA at a specific recognition sequence, known as the E-box (5′-CANNTG-3′). Among 133 bHLH genes described initially in *Arabidopsis thaliana*, 113 were shown by reverse-transcription polymerase chain reaction (RT-PCR) to be expressed in at least one out of 12 tissues or conditions tested, most of them showing a broad expression pattern, and only two exhibited a root specific expression (Heim *et al.*, 2003). In legumes, no bHLH survey has yet been published, although > 100 bHLH sequences are present in the *M. truncatula* gene atlas data base (MtGEA) (Benedito *et al.*, 2008). Only two legume *bHLH* genes associated with root development or nodulation have been studied so far: *GmSAT*, originally described as encoding a soybean ammonium transporter (Kaiser *et al.*, 1998; Marini *et al.*, 2000), and *LjRHL1* involved in *L. japonicus* root hair development (Karas *et al.*, 2009).

Here we present the characterization of a *M. truncatula bHLH* gene, *MtbHLH1*, which is specifically expressed in roots and nodules. Based upon various functional data we propose that this gene is involved in nodule vasculature patterning and in the control of nutrient exchange between nodules and the rest of the plant.

## Materials and Methods

### Plant growth, bacterial strains

*Medicago truncatula* Gaertn. cv Jemalong A17 was used as the wild-type reference for all the experiments. Surface-sterilized seeds were placed on inverted agar plates in the dark for 3 d at 8°C and 1 d at 20°C. For hormone and Nod factor (NF) treatments, germinated seeds were grown on Farhaeus medium agar plates covered with growth pouch paper, at 25°C with a photoperiod of 16 h light: 8 h dark.

Following transformation, composite plants with transgenic roots were transferred in growth pouches for rhizobial inoculations (Vernié*et al.*, 2008). For root phenotype studies they were transferred onto 21 cm^2^ Farhaeus agar plates containing 1 mM NH_4_NO_3_ (to avoid N starvation), covered with growth pouch paper. Wild-type *S. meliloti* RCR2011 pXLGD4 (GMI6526) and *S. meliloti* RCR2011 *exoA* pXLGD4 (GMI3072) were grown as described by Vernié*et al.* (2008).

### Hormone and Nod factor treatments

10 mM stock solutions were prepared in 0.1 M KOH for benzyl-amino-purine (BAP), and in 50% water-50% ethanol for IAA, ABA (Sigma-Aldrich) and purified NF from *S. meliloti*. The 10 μM hormone and 1 nM NF working solutions were then prepared in water. Aliquots of 2 ml of these solutions were applied with a pipette onto roots of 10 5-d-old A17 seedlings, placed on Farhaeus agar plates covered with growth pouch paper. Thirty roots per time-point (0, 2, 4, 8 and 24 h after treatment) were cut and frozen before RNA extraction. Three biological repetitions were done for each of these treatments. RNA extraction and quantitative (q)RT-PCR were performed as described in Combier *et al.*, 2006).

### Plasmid constructs and *A. rhizogenes* transformation

The T3 primer and two nested primers 5′-CAATCTTCATAAGTTGTCCTGG-3′ and 5′-GGTAATTGTGTTGTTCCATTGTG-3′ designed from the initial 601 bp SSH (suppression subtractive hybridization) fragment, MtD19113 (Godiard *et al.*, 2007) were used to amplify the lacking 5′ cDNA region by primer extension in a lambda-Zap cDNA library of *M. truncatula* 4-d-old nodules (Gamas *et al.*, 1996). A 921 bp DNA fragment overlapping 176 bp of the initial SSH DNA fragment was cloned and sequenced, resulting in a full size 1389 bp *MtbHLH1* cDNA fragment.

To generate the *P35S::MtbHLH1-EAR* construct, we first introduced the EAR domain in the pPex vector (Combier *et al.*, 2006). The EAR domain was obtained by fusing two oligonucleotides corresponding to the EAR sequence (Hiratsu *et al.*, 2003) carrying a *Bam*HI and a *Xba*I restriction sites at the 5′ and 3′ site, respectively: 5′-GATCCCTTGATCTGGACCTAGAATTGAGACTTGGATTCGCT-3′ and 5′-CTAGAGCGAATCCAAGTCTCATTACTAGGTCCAGATCAAGG-3′ (Invitrogen). The resulting DNA fragment was introduced in the pPex vector between *Bam*HI and *Xba*I sites, resulting in the pPex-EAR vector. We amplified the complete *MtbHLH1* coding sequence from the nodule cDNA library (Gamas *et al.*, 1996) using Pfx polymerase (Invitrogen) and primers 5′-TACTCGAGATGGCTCTTGAAACTGTGG-3′ and 5′-GCGGATCCATTTAGTTGATAAGCCAGTTC-3′ and inserted it into pPex-EAR between the *Xho*I and *Bam*HI sites. We checked by sequencing that the *MtbHLH1* coding sequence was fused in frame with the 12 amino acids LDLDLELRLGFA of the EAR sequence, as required for CRES-T. Finally, we added into this plasmid, at its KpnI site, the *DsRED* expression cassette from the pRed Root vector (Limpens *et al.*, 2004). The pPex vector carrying the same *DsRED* construct was used as control in all the transformation experiments.

To generate the *PMtbHLH1::GUS* construct, we amplified a 1463-bp fragment (3903-5365) from the MTH2-155M7 genomic BAC clone using Pfx polymerase and primers 5′-CGGGGTACCACCGTGTTCACGAACGAGAT-3′ and 5′-CATGCCATGGTATTATTATTAATTTGTGACTAATC-3′ and inserted it between the *Kpn*I and *Nco*I sites of the pPex-GUS vector (Combier *et al.*, 2006).

All the constructs were checked by sequencing, introduced into *A. rhizogenes* strain *ARqua1* by electroporation and used for *M. truncatula* root transformation (Boisson-Dernier *et al.*, 2001). Transgenic roots were selected on Farhaeus agar plates supplemented with 25 μg ml^−1^ kanamycin and were checked for fluorescence resulting from the expression of a DsRed gene present on the T-DNA. The DsRed negative roots were eliminated as soon as they were detected.

### Histochemical staining and microscopy studies

Histochemical glucuronidase (GUS) staining (using X-Gluc, 5-bromo-4-chloro-3-indolyl-β-glucuronic acid; MP Biomedicals, Europe, Illkirch, France), preparation and observation of nodule or root sections (100 or 50 μm), or thinner sections (10 μm) embedded in Technovit 7100 resin, were performed as described in Combier *et al.*, (2007).

Before clearing with Hoyer's solution (Bougourd *et al.*, 2000), the nodules were detached from roots and fixed in a 1.5% glutaraldehyde solution in phosphate buffer 0.1 M, pH 7, rinsed three times in the same buffer and briefly (< 1 min) treated with 1% sodium hypochlorite until the nodule cell walls became translucent, and finally washed three times in water. They were placed on a glass slide, superficially dried with pure ethanol and rapidly immerged in the Hoyer's solution. The cleared entire nodule content could be observed 3 d later.

### Microarray studies and qRT-PCR analyses

RNA was extracted and amplified from *P35S::MtbHLH1-EAR* and control nodules as previously described (Vernié*et al.*, 2008). Mt16KOLIPlus microarray hybridizations and analyses were performed as indicated in Vernié*et al.* (2008). Validation of microarray results were performed by qRT-PCR on 384-well plates with a Lightcycler LC480 (Roche) using first-strand cDNA obtained from 500 ng of nonamplified total RNA extracted at 24 d post-inoculation (dpi) from either *P35S::MtbHLH1-EAR* or control pPex-DsRed isolated nodules, from three independent experiments. The primers used (Supporting Information Table S1) were designed with primer express v2.0 Software (Applied Biosystems France, Sainte Geneviève des Bois, France).

### Accession numbers

All data files for Mt16KOLIPlus microarrays are available through the ArrayExpress database (ArrayExpress; http://www.ebi.ac.uk/arrayexpress/; array accession number E-TABM-719). The *MtbHLH1* gene name and sequence have been registered at the Genbank database (accession number FR697055).

## Results

### *MtbHLH1*-encoded protein has a typical bHLH transcription factor domain

*MtbHLH1* was initially identified from a SSH cDNA library made from whole-root systems of the supernodulating *M. truncatula sunn-2* mutant inoculated with *S. meliloti.* The corresponding expressed sequences tag (EST) (termed MtD19113) was found to encode a bHLH transcription factor domain, and shown to be upregulated in *M. truncatula* 4-, 10- and 14-d-old nodules (Godiard *et al.*, 2007). We found with the Legoo knowledge data base (http://www.legoo.org) that MtD19113 corresponds to MTGI7-TC84416, described to be upregulated by *S. meliloti* as early as 12 h post-inoculation (Lohar *et al.*, 2006), and to Mtr.10993.1.S1_at, that shows a maximal expression in roots and nodules among a large range of tested organs and conditions (MtGEA, Benedito *et al.*, 2008).

The 5′ cDNA region, absent from the MtD19113 EST clone, was amplified by primer extension from a cDNA library (see the Materials and Methods section). The resulting 1389 bp cDNA fragment corresponded to the size of the *MtbHLH1* mRNA detected by Northern blot (data not shown) and was thus considered to be full size. This transcript is predicted to encode a 321 amino acid protein, showing the typical basic helix–loop–helix motif found in plant bHLH proteins (Carretero-Paulet *et al.*, 2010) at amino acids 121 to 181 (Fig. S1). In the basic region predicted to bind DNA, MtbHLH1 has five basic amino acids and His-Glu-Arg-Arg (H-E-R-R) residues at positions 9, 13, 16 and 17 shown to be critical for DNA binding in several bHLH proteins (Brownlie *et al.*, 1997). These conserved residues classify MtbHLH1 as a putative G-box (5′-CACGTG-3′) binder, which is a specific type of E-box (Brownlie *et al.*, 1997; Atchley *et al.*, 1999; Carretero-Paulet *et al.*, 2010). Highly hydrophobic residues are conserved in helix 1 and 2 at every position reported to be involved in protein–protein interactions, including notably the Leu27 residue in helix 1 and the Leu73 in helix 2, which are reported to be required in the dimerization process and in DNA–protein complex stability (Brownlie *et al.*, 1997; Massari & Murre, 2000).

The MtbHLH1 bHLH domain therefore fulfils the consensus sequence criteria and contains all the amino acid residues described as important for DNA binding or protein–protein interactions, suggesting that MtbHLH1 is likely to be functional as a bHLH TF.

To take advantage of functional information available for some of the 155 described *A. thaliana bHLH* genes (Heim *et al.*, 2003; Toledo-Ortiz *et al.*, 2003), the *A. thaliana* bHLH proteins exhibiting the highest conservation with MtbHLH1 were searched. The best score was obtained with AtbHLH096 bHLH protein (AT1G72210), which presents 48% identity, and 61% similarity with MtbHLH1 (expected value = 2e-76) (Fig. S1). An alignment of both protein sequences shows that the best conserved regions are the bHLH motif and adjacent amino acids as well as a region of *c.* 80 amino acids near the *C*-terminus. An alignment of the *MtbHLH1* cDNA and genomic (Medtr3g150170.1) sequences indicated that the *MtbHLH1* gene has two introns of, respectively, 892 bp and 99 bp, the position of which is conserved in *AtbHLH096* gene (Fig. S1). The expression data available for *AtbHLH096* reveal that it is transcribed in many conditions and organs, including roots (Heim *et al.*, 2003). More detailed functional data are available for another closely related AtbHLH protein, FAMA (=AtbHLH097; 43% identity, 54% similarity) which is required in the first cell divisions establishing the stomatal guard cell lineage (Ohashi-Ito & Bergmann, 2006; MacAlister *et al.*, 2007).

### *MtbHLH1* gene is expressed in roots and induced by auxin treatment

**Fig. 1:**
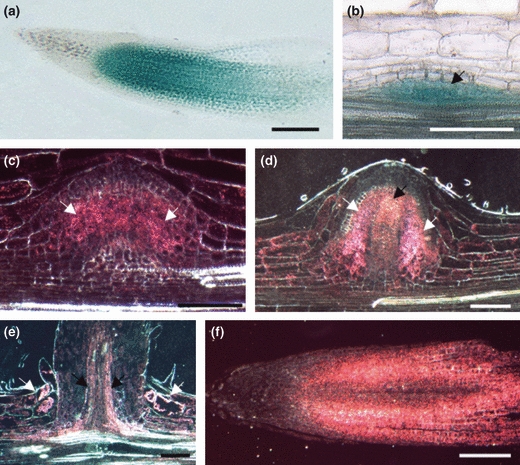
Localization of *MtbHLH1* gene expression in *Medicago truncatula* roots. (a–f) Localization of *PMtbHLH1::GUS* activity in root tips (a), in dividing pericycle cells (arrow) of lateral root primordia (b), in lateral root primordium cells derived from pericycle cells (arrows) but not in cells derived from cortical cells (c), at the top (d, black arrow) and in the region (white arrows) surrounding the differentiating root vascular bundle of emerging lateral root (d), in the pericycle layer delimiting the central vascular tissue of elongated lateral roots (e, black arrows) and in the cortical cells of the main root at the site of emergence of the lateral root (e, white arrows), and in several cell layers in the root meristematic zone (f). (a,b) Root whole-mount observations, glucuronidase (GUS) activity in blue. (b–f) Thin root sections (5 μm) embedded in Technovit before GUS staining, dark-field observations, GUS activity in pink/red. Bars, 100 μm.

To precisely determine the tissue localization of *MtbHLH1* transcripts, we generated a transcriptional fusion between a 1.44 kbp *MtbHLH1* promoter fragment and the *GUS* reporter gene. The expression pattern of this *PMtbHLH1::GUS* construct was examined in *A. rhizogenes*-transformed *M. truncatula* roots. The highest nonsymbiotic expression was found in the root meristematic region, with a signal strongly diminishing in the root elongation zone (Fig. 1a). *MtbHLH1* expression was also detected in lateral root primordia, where it was first confined to the dividing pericycle cells while it was undetectable in the adjacent endodermis or cortical cell layers (Fig. 1b). At a later stage of development, most internal root primordium cells were intensely stained for GUS activity (Fig. 1c). A closer observation revealed that GUS expression was found in primordium cells derived from pericycle cells but not in cells derived from cortical cells (Fig. 1c). When the lateral root began to emerge, GUS staining was largely restricted to the regions at the top and surrounding the differentiating root vascular bundle (Fig. 1d). On elongated lateral roots, GUS activity was observed in the pericycle layer delimiting the central vascular tissue (Fig. 1e) and in the cortical cells of the main root at the site of emergence of the lateral root (Fig. 1e, arrowheads). Finally, GUS staining was also detected in several cell layers in the lateral root meristematic zone (Fig. 1f), as in the main root (Fig. 1a).

**Fig. 2:**
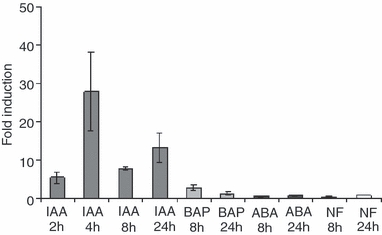
Quantitative reverse-transcription polymerase chain reaction analyses of (*Medicago truncatula*) *MtbHLH1 gene expression* after hormonal or Nod factor (NF) treatment**.** Treatments with, respectively, 10 μM Auxin (IAA), an analogue of cytokinin, benzyl-amino-purine (BAP), ABA or purified NF (10^−9^ M). Results show the fold induction compared with a mock treatment with water. Mean values ±SE are given from three independent experiments.

Such an expression pattern is reminiscent of that exhibited by auxin-induced genes, notably in *M. truncatula* (van Noorden *et al.*, 2007; Mathesius, 2008). We thus decided to test whether *MtbHLH1* expression could be upregulated by exogenous application of IAA on *M. truncatula* roots. A qRT-PCR analysis revealed that *MtbHLH1* was indeed significantly induced (fivefold) as early as 2 h after 10 μM IAA treatment, while maximum induction (28-fold) was reached 4 h post-treatment (Fig. 2). The emergence of numerous IAA-induced lateral roots was observed *c.* 3 d after *MtbHLH1* induction by IAA. Similar experiments conducted with an analogue of cytokinin, BAP, ABA (both at 10 μM) or purified Nod factors (NF, 10^−9^ M) did not show any strong and reproducible *MtbHLH1* induction.

### *MtbHLH1* is expressed in nodule vascular bundles and in uninfected cells of the fixation zone from early to late nodulation stages

**Fig. 3:**
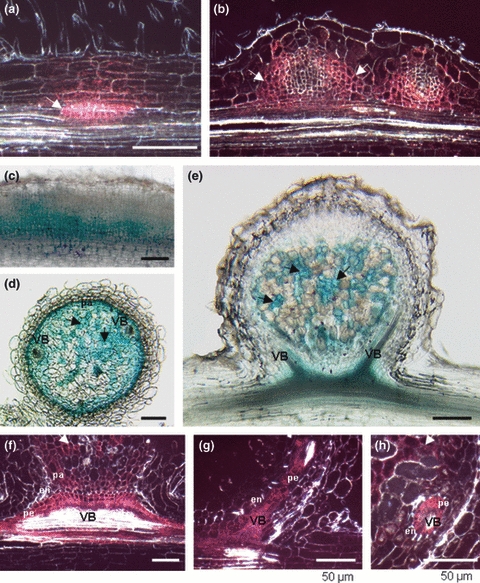
Localization of *MtbHLH1* gene expression in *Medicago truncatula* nodules. (a–h) Localization of *PMtbHLH1::GUS* activity in dividing pericycle and endodermis cells of young nodule primordia (a, arrows), in the peripheral cell layers of the developing nodule (b, arrows), in 6-d-old nodules induced by an infection-defective *exoA* mutant of *Sinorhizobium meliloti* (c), in vascular bundles (VB), peripheral parenchyma (pa) and uninfected cells (arrows) of fixation zone III of mature 24-d-old nodules as shown in transverse (d) and longitudinal sections (e–h), and more precisely, in the cell layers surrounding the VBs at the nodule base (f), and in the pericycle cell layers (pe) of nodule VBs, but completely absent from the root and nodule endodermis (en) (g,h). (a,b,f–h) Thin root sections (5 μm) embedded in Technovit before GUS staining, dark-field observations, GUS activity in pink/red. (c–e) 100-μm thick root sections, GUS activity in blue. Bars: (a–f) 100 μm; (g,h) 50 μm.

Upon inoculation by wild type (WT) *S. meliloti*, *PMtbHLH1::GUS* activity was detected in the dividing pericycle and endodermis cell layers of young nodule primordia, while a lower expression was revealed in the adjacent cortical cells (Fig. 3a). During nodule primordium growth, GUS staining was then restricted to the peripheral cell layers of the developing nodule (Fig. 3b). Indeed, at this stage, no expression was detected in the nodule central zone infected by *S. meliloti* bacteria (Fig. 3b), or in infected root hairs (data not shown), indicating that *MtbHLH1* expression is not associated with bacterial infection. We then examined empty nodules induced by an infection-defective *exoA* mutant of *S. meliloti* (Yang *et al.*, 1994). A very clear *PMtbHLH1::GUS* expression was detected in 6-d-old *exoA* nodules (Fig. 3c), while *MtbHLH1* induction was found to be statistically significant in 10-d-old *exoA* nodules by microarray analysis (adjusted *P-*value of 0.04, Moreau *et al.*, 2011), confirming that *MtbHLH1* expression takes place during nodule development but is not dependent upon *S. meliloti* infection *per se*.

In longitudinal sections of fully developed nodules (24 dpi, Fig. 3e), *PMtbHLH1::GUS* expression was absent from the meristematic zone I and infection zone II but was detected in nodule VBs and fixation zone III. Interestingly both longitudinal (Fig. 3e) and transverse (Fig. 3d) nodule sections revealed that GUS expression was only detected in the uninfected cells (UCs). In addition, observations of nodule transverse sections showed that *PMtbHLH1::GUS* was expressed in VBs, in the nodule peripheral parenchyma and in a network of UCs that seemed to be connected to the VBs (Fig. 3d). Longitudinal sections also revealed a strong GUS expression at the nodule base, in the region connecting the nodule to the root VB, particularly in the cell layers surrounding the VBs (Fig. 3f). Closer observations indicated that *PMtbHLH1::GUS* expression was restricted to the pericycle cell layers of nodule VBs, and completely absent from the root and nodule endodermis (Fig. 3g,h).

### Transgenic roots expressing CRES-T impaired *MtbHLH1* fusions produce nitrogen-fixing nodules with vascular defects

As the search for *MtbHLH1* mutants in *M. truncatula* Tilling populations was unsuccessful, we decided to use the CRES-T approach (Chimeric REpressor Silencing Technology, Hiratsu *et al.*, 2003) to investigate the role of MtbHLH1. The principle of this approach is to fuse a 12 amino acid repression domain (EAR domain) to the transcription factor tested, thus allowing the transcription of its specific target genes to be suppressed, even in the presence of functionally redundant transcription factors. The CRES-T approach has been used successfully in *A. thaliana* to convert various TF types into dominant repressors and to study their function *in planta* (Matsui *et al.*, 2005; Koyama *et al.*, 2007; Mitsuda *et al.*, 2007). We expressed the *MtbHLH1::EAR* fusion under the control of the CaMV 35S promoter, whose expression pattern in nodules is particularly well suited for *MtbHLH1* reverse genetics studies*.* Indeed, Auriac & Timmers (2007) have shown that the P35S::GUS leads to a high level of GUS expression in all root tissues and in most nodule cells except the meristematic zone and the invaded cells of zone III (tissues where no *PMtbHLH1::GUS* expression was found). Importantly, this study indicated a high level of P35S activity in the nodule vascular system and in uninvaded cells of zone III, that is, the tissues showing maximal *MtbHLH1* expression*.*

Uninoculated *P35S::MtbHLH1-EAR* transgenic roots grown in the presence of N (1 mM ammonium nitrate) did not show developmental or growth defects compared with control transgenic roots (empty vector-transformed). They actually showed a statistically significant increase in the total number of roots per plant (Welch *t*-test *P*-value = 0.0038; mean = 21 ± 1.44 (standard error) (*n* = 56) for *P35S::MtbHLH1-EAR* roots vs 15 ± 1.18 (*n* = 50) for control roots, 14 d after selection of transformed roots (dps)). They also showed an increase in growth, as estimated by the longest root length (Welch *t*-test *P*-value = 6.234e-05; mean = 8.60 cm ± 0.54 (*n* = 56) for *P35S::MtbHLH1-EAR* roots vs 5.84 ± 0.38 (*n* = 50) for control roots) (see box plots in Fig. S2). The dry weight of the corresponding aerial parts was also determined for 14 dps and 16 dps plants and did not show a statistically significant difference (Mann–Whitney test, *n* = 16, 14 dps and *n* = 19, 16 dps).

**Fig. 4:**
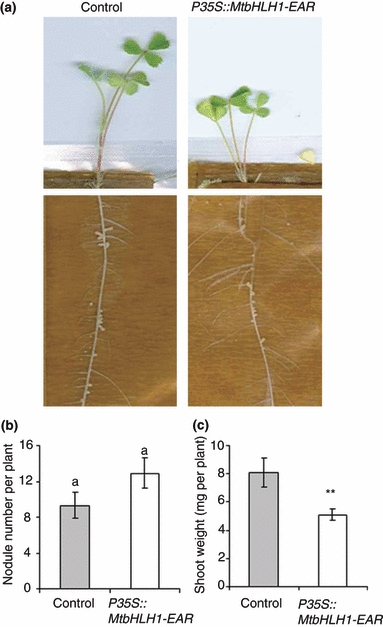
Morphology of composite *Medicago truncatula* plants bearing transgenic roots expressing a CRES-T impaired MtbHLH1 fusion, after inoculation with *Sinorhizobium meliloti.* (a) Corresponding aerial untransformed plant parts (upper panels) and nodulated roots (lower panels) of *P35S::MtbHLH1-EAR* transformed roots (right) showing smaller nodules and aerial part compared with control empty vector-transformed roots (left), at 24 d post-inoculation (dpi). (b) Mean nodule number per plant (24 dpi) was not significantly different in *P35S::MtbHLH1-EAR* compared with control transformed roots (Mann–Whitney test), but (c) the mean dry weight of the aerial parts (16 dpi) was significantly affected (Mann–Whitney test, **, *P* < 0.01). Mean values ±SE are given from three independent transformation experiments.

Upon *S. meliloti* root inoculation, the nodules produced on *P35S::MtbHLH1-EAR* transgenic roots appeared, on average, with a delay of 3 d compared with transformed control roots (8.7 ± 2.7 dpi to obtain 50% of nodulated plants compared with 5.7 ± 1.2 dpi in transformed control plants) and remained generally smaller than control nodules, even though some of them became elongated and clearly pink, which was indicative of the synthesis of leghaemoglobin (Fig. 4a). The number of nodules produced on *P35S::MtbHLH1-EAR* transgenic roots was not significantly different from the number observed in transformed control roots at 24 dpi (Mann–Whitney test, *n* = 28 plants) (Fig. 4b). However the untransformed aerial parts of *P35S::MtbHLH1-EAR* nodulated plants were not as vigorous as control transformed plants (Fig. 4a) and exhibited a 40% reduction in dry weight (statistically significant in a Mann–Whitney test, *P* < 0.01, *n* = 15) (Fig. 4c). Interestingly, this was not caused by an inability of *P35S::MtbHLH1-EAR* nodules to fix N as they were Fix+, as determined by an acetylene reduction assay performed on nodulated plants at 21 dpi (production of 78.3 ± 9.2 ppm ethylene per plant per h compared with 31.9 ± 8.2 ppm for control plants, *n* = 8 plants). Moreover electron microscopy observation of bacteroids from the *P35S::MtbHLH1-EAR* nodule fixation zone showed normal type IV bacteroids, as in control plants (data not shown). The fact that the plant aerial part could not fully benefit from symbiotic N fixation taking place in *P35S::MtbHLH1-EAR* nodules suggested a defect in the transfer of reduced N from nodules to the rest of the plant.

**Fig. 5:**
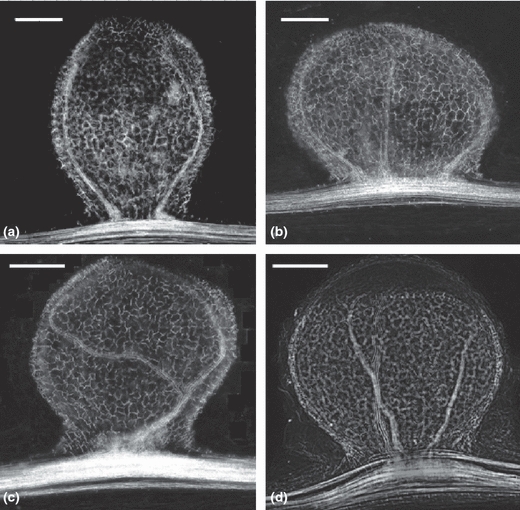
Nodule vascular architecture in P35S::MtbHLH1-EAR transformed *Medicago truncatula* roots. (a,b) Nodules from empty vector transformed control plants (24 d post-inoculation (dpi)) and (c,d) nodules from *P35S::MtbHLH1-EAR* transformed plants (24 dpi). (a–d) Whole-mount nodules cleared with Hoyer's solution, dark field observations. Bars, 200 μm.

**Fig. 6:**
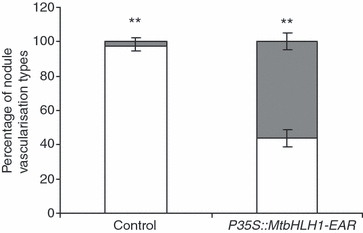
Quantification of normal and abnormal vascular architecture of nodules. The percentage of each vascular type was calculated from microscope pictures of 68 control transformed and 42 *P35S::MtbHLH1-EAR* nodules, obtained in four independent transformations of 10–12 different *Medicago truncatula* plants. **, Statistically significantly different values (Mann–Whitney test, *P* < 0.01). Open and closed, respectively normal and abnormal vascular bundle organization.

We then investigated whether the *P35S::MtbHLH1-EAR* nodule structure was normal by examining longitudinal sections. Surprisingly, this revealed frequent alterations in the VB structure, which exhibited a nonsymmetrical organization rarely observed in control nodules. By contrast, uninoculated roots did not reveal any difference with control roots in VB organization, as determined from semi-thin longitudinal and cross-sections of roots by light microscopy (data not shown). To be able to observe the whole vascular system, which is not possible with nodule sections, we cleared the cell content from whole nodules with Hoyer's solution (Bougourd *et al.*, 2000), which allowed the lignified cell walls and VBs to be visualized. This method considerably facilitated the comparative analysis of the VB structure of *P35S::MtbHLH1-EAR* and control nodules. Two main organization types were observed: (1) A normal organization with generally two opposite VBs showing well separated connections with the root VB, with angles to the root VB close to 60° (Fig. 5a,b); an additional VB was sometimes visible in a perpendicular plane to the first ones (Fig. 5b) and in all cases nodule VBs followed the nodule outer cortex borders. (2) An abnormal organization, with either a single, generally branched, VB (Fig. 5c) or two to three VBs that originate very close to each other at the base of the nodule (Fig. 5d), with variable angles to the root VB and variable growth patterns (Fig. 5c,d). The first type was found in 97% of transformed control nodules (*n* = 68, 15 plants), compared with only 44% in *P35S::MtbHLH1-EAR* nodules (*n* = 42, 12 plants); conversely the second type was frequent (56%) in *P35S::MtbHLH1-EAR* nodules but rare (3%) in control nodules (Fig. 6). This result suggests that impairing MtbHLH1 function leads to an alteration of the developmental pattern of nodule VBs.

### Possible targets of the MtbHLH1 Transcription Factor revealed by transcriptome analyses

**Fig. 7:**
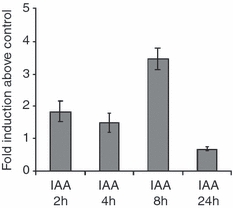
Quantitative reverse-transcription polymerase chain reaction analysis of *MtLOB-AS2like* (*Medicago truncatula*) expression after auxin IAA treatment (10 μM). Mean values ±SE are given from three independent experiments.

To look for possible direct or indirect MtbHLH1 target genes in nodules, we performed microarray analyses of isolated nodules harvested at 24 dpi from *P35S::MtbHLH1-EAR* or control vector transformed roots. We used Mt16KOLI1Plus microarrays carrying 16 470 *M. truncatula* 70-mer oligonucleotide gene probes (Kuster *et al.*, 2007). Genes were considered as candidate differentially expressed genes when the difference in expression showed a *P*-value < 0.001, in three independent experiments. Only a small number of candidate genes was found, with 24 downregulated and seven upregulated in *P35S::MtbHLH1-EAR* compared with control nodules (Ratio (*R*) > 1.5, Table S2). Among these seven gene probes were two 70-mer oligonucleotides corresponding to different parts of the *MtbHLH1* gene itself (MT012710, *R* = 4.8 and MT013931, *R* = 5.7), indicating its overexpression in *P35S::MtbHLH1-EAR*, as expected. Twelve candidate differentially expressed genes (11 downregulated and one upregulated) showing potentially interesting homologies were then tested by qRT-PCR and confirmed for differential expression (Table 1). The genes validated as downregulated in *P35S::MtbHLH1-EAR* comprised a set of genes associated with the differentiation of nitrogen-fixing nodules, namely a leghaemoglobins protein (LGB2), two late nodulins, a nodule-cysteine-rich protein (NCR072) (Mergaert *et al.*, 2003) and a glycine-rich protein (GRP3B) (Kevei *et al.*, 2002). They also included genes encoding an asparagine synthase, which catalyses the synthesis of the primary product of N assimilation in *Medicago* (Shi *et al.*, 1997), an ABC transporter, an unknown function BURP domain protein and a putative transcription factor (gene probe MT000746). The last of these belongs to the lateral organ boundaries domain (LOB or LBD) protein family (Shuai *et al.*, 2002). This *M. truncatula* sequence, represented by Mtr.4306.1.S1_s_at in the MtGEA, encodes a typical class II LOB protein with a CNGCRVLRKGCSENC consensus sequence and a Pro111 conserved residue (Shuai *et al.*, 2002). It is highly homologous (55% identity, 88% similarity) to the *A. thaliana* ASYMMETRIC LEAVES 2-like protein (AS2, also called LBD41) (Semiarti *et al.*, 2001). We then tested by qRT-PCR whether this *MtLOB-AS2like* gene could be induced in *M. truncatula* roots treated by IAA, similarly to *MtbHLH1*. We found that *MtLOB-AS2like* was indeed induced by auxin with a maximal induction ratio of 3.5 at 8 h post-treatment, 4 h after the observed maximal induction of *MtbHLH1* (Fig. 7). This was consistent with a possible control of *MtLOB-AS2like* by MtbHLH1 TF.

**Table 1:** Putative target genes of the MtbHLH1 transcription factor

			Relative expression		Relative expression		
						
			Mt16KOLIPlus microarrays		qRT-PCR	Boxes in promoter (2) as index	
					
Reporter	Affymetrix ID	Annotation	*P*-value	P35S::MtbHLH1-EAR/Control nodule^1^	P35S::MtbHLH1-EAR/Control nodule ± SE^1^	E-box	G-box
MT014172	Mtr.40134.1.S1_x_at	Leghaemoglobin 2	0.00072	0.46	0.332 ± 0.123	2	0
MT009727	Mtr.9019.1.S1_at	NCR072	0.00006	0.66	0.553 ± 0.195	nd	nd
MT012042	Mtr.3236.1.S1_at	Late nodulin	0.00001	0.58	0.396 ± 0.119	nd	nd
MT003243	Mtr.13105.1.S1_at	Late nodulin	0.00010	0.58	0.297 ± 0.113	3	1
MT003190	Msa.955.1.S1_x_at	Nodule-specific GRP-repeat	0.00004	0.66	0.576 ± 0.136	7	0
MT000131	Mtr.8499.1.S1_at	Asparagine synthase	0.00004	0.59	0.29 ± 0.026	4	1
MT000197	Mtr.37357.1.S1_at	E-class P450, group I	0.00002	0.81	0.562 ± 0.127	2	0
MT015543	Mtr.10732.1.S1_at	Wound-responsive family protein	0.00016	0.64	0.469 ± 0.101	nd	nd
MT009942	Mtr.38312.1.S1_at	ABC-2 type transporter	0.00020	0.56	0.236 ± 0.066	nd	nd
MT000746	Mtr.4306.1.S1_s_at	LOB domain protein	0.00011	0.69	0.474 ± 0.034	nd	nd
MT007140	Mtr.8505.1.S1_at	BURP domain protein	0.00005	0.60	0.378 ± 0.088	1	0
MT000263	Mtr.48601.1.S1_at	BURP domain protein	0.00007	1.99	2.383 ± 0.838	3	0

qRT-PCR, quantitative reverse-transcription polymerase chain reaction.

1 Expression ratios calculated between *P35S::MtbHLH1-EAR* and control nodules either from microarray or qRT-PCR from three independent experiments.

^2^Number of palindromic E- and G-boxes in the promoter region of the corresponding genes (600 bp for E-boxes, 1500 bp for G-boxes).

Having a promoter sequence available in seven out of the 12 validated putative MtbHLH target genes, we searched for possible E-boxes (5′-CANNTG-3′), and especially the palindromic ones, or G-boxes (5′-CACGTG-3′), described to be bound by bHLH proteins in, respectively, the 600 bp or the 1500 bp fragment upstream of the coding sequence start (Table 1). All promoter fragments tested have one to seven palindromic E-boxes and two of them, corresponding, respectively, to the late nodulin Mtr.13105.1.S1_at and to the asparagine synthase gene, carry an additional G-box, suggesting that they might be direct target genes of MtbHLH1.

## Discussion

The bHLH family is one of the largest transcription factor families in plants, with members described to be involved in a variety of signalling and developmental processes (Carretero-Paulet *et al.*, 2010). No bHLH gene family has yet been analysed in legumes, whereas this has been done for several other plants whose genome is fully sequenced such as Arabidopsis, poplar, rice and moss (*Physcomitrella patens*) and five algae (Heim *et al.*, 2003; Toledo-Ortiz *et al.*, 2003; Li *et al.*, 2006; Carretero-Paulet *et al.*, 2010). Yet, until now, very few bHLH genes have been characterized in legumes and found to play a role in symbiotic interactions (Kaiser *et al.*, 1998; Libault *et al.*, 2009). Here we describe a *M. truncatula* gene, *MtbHLH1*, with such a function and which encodes a protein exhibiting the hallmarks of bHLH proteins in its predicted DNA binding and protein–protein interaction region. The closest *A. thaliana* homologues of MtbHLH1, such as AtbHLH096, belong to one of the 12 land plant-specific bHLH subfamilies (32 subfamilies in total), which greatly expanded after the split between green algae and land plant species, and led to the establishment of most of the diversity of DNA-binding and protein motifs of plant bHLH proteins (Carretero-Paulet *et al.*, 2010).

The cellular expression pattern of *MtbHLH1* is quite unusual among the nodule-induced genes that have been studied to date. *MtbHLH1* is expressed in the VB pericycle cells from early to late nodulation stages, and it is also expressed in the UCs of the fixation zone III. Finally *MtbHLH1* is induced in the root by external auxin treatment while its expression pattern as determined by promoter::GUS fusion analysis is consistent with auxin-induction in roots. *MtbHLH1* expression is first observed in pericycle cells of lateral roots and nodule primordia, which represent the cells where initiation of these organs takes place (Timmers *et al.*, 1999). Nodule organogenesis and lateral root formation are similar in the sense that they both require auxin for primordia establishment and vasculature differentiation (de Billy *et al.*, 2001; Mathesius, 2008). When MtbHLH1 was fused to the EAR repressor domain (CRES-T approach (Hiratsu *et al.*, 2003)), we observed two nodulation phenotypes: a modified patterning of nodule VB development, with most VBs having a unique or two to three VBs originating very close to each other instead of two or three well-spaced VBs as in WT nodules; and a reduced growth of aerial parts of the plant, even though *P35S::MtbHLH1-EAR* nodules were able to fix atmospheric N.

In view of the *MtbHLH1* expression pattern in roots, it is intriguing that we did not detect defects in root growth and development (including VB structure) in *P35S::MtbHLH1-EAR* roots in the presence of exogenous N*.* By contrast, root growth even seemed to be improved by this construct. However, the fact that a same construct has distinct or opposite effects in root and nodule development has already been observed with several genes, notably the auxin-related *M. truncatula* CDC16 gene (Kuppusamy *et al.*, 2009). The role or the mechanism of action of MtbHLH1 is thus likely to be different in roots and in nodules. For example MtbHLH1 could act within transcriptional repressor complexes in roots, which might not be altered by a fusion to the EAR repressor domain, vs transcriptional activator complexes in nodules. Indeed, MtbHLH1 is likely to interact with other bHLH or TF proteins, like many members of the bHLH family (Ramsay & Glover, 2005), the nature of which may depend on whether a root or a nodule is being formed. In any case, the absence of root defects in plants bearing the *P35S::MtbHLH1-EAR* construct indicates that the reduced plant growth observed under symbiotic conditions is very likely caused by altered nodule structure and/or function.

Many bHLH proteins are involved in the formation of different lateral organs in plants (Takeda *et al.*, 2003; Gallavotti *et al.*, 2004). Studies on two Arabidopsis mutants perturbed in pericycle differentiation, *lonesome highway* (*lhw*) and *impaired vasculature development* (*ivad*), have revealed an intimate correlation between pericycle cell fate and vascular organization of the nascent root. *lhw* encodes a noncanonical bHLH TF able to interact with different bHLH proteins (Ohashi-Ito & Bergmann, 2007) and is required to establish the normal diarch pattern of root vasculature tissue (Parizot *et al.*, 2008). The *P35S::MtbHLH1-EAR* nodule phenotype suggests that MtbHLH1 is involved in determining the symmetrical organization of nodule vasculature, and the delay of nodule appearance observed at early time-points could be a consequence of an altered vascularization.

The development of nodule vascularization is poorly documented and MtbHLH1 is one of the first TF described to affect this process. Imaizumi-Anraku *et al.* (2000) have described a *L. japonicus* fix^–^ mutant, *alb1*, that forms empty nodules with only one VB differentiating at the proximal nodule end, whereas in WT *L. japonicus* nodules VBs bifurcate from the root stele to surround the central infected zone. The gene corresponding to the *alb1* mutation has not been identified but the *alb1* phenotype appears more severe and broader than the one observed in *P35S::MtbHLH1-EAR* nodules, with no bacterial release and poor *ENOD40* induction (Yano *et al.*, 2006).

More mutants affected in VB formation or patterning have been described in leaves and roots, the characterization of which has shown a major role played by auxin-related genes (for reviews see Rolland-Lagan, 2008; Peret *et al.*, 2009). The so-called canalization hypothesis proposed for leaf veins, states that auxin transport through cells promotes their differentiation into veins and thereby increases their capacity to transport auxin. It is very likely that patterning of nodule VBs also involves local auxin fluxes, and it should be recalled that expression of *MtLAX* genes encoding auxin influx proteins correlates with VB formation in nodule primordia (de Billy *et al.*, 2001). MtbHLH1 may therefore be involved in the localization of auxin maxima or in auxin-regulated events. The position of auxin maxima probably varies depending on whether a root or a nodule is being formed, leading either to a single central VB (in roots) or several peripheral VBs (in nodules). *MtbHLH1* expression is efficiently induced by auxin but we have no evidence that *MtbHLH1* controls auxin transporters and thereby contributes to a positive feedback loop as proposed in the canalization hypothesis. However, we found that MtbHLH1 controls the expression of a gene encoding a protein very similar to the LOB transcription factor AS2 described to regulate leaf venation. Thus, in *A. thaliana*, an *as2* mutant exhibits asymmetrical venation and disconnected or insufficiently connected veins (Semiarti *et al.*, 2001), while ectopic AS2 expression leads to an altered vein patterning (Lin *et al.*, 2003). Moreover Zgurski *et al.* (2005) have shown that the *as2* phenotype is correlated with asymmetric auxin response. The *MtLOB-AS2*like gene thus represents an attractive candidate to mediate MtbHLH1 role on VB development. Interestingly, the *A. thaliana LOB* gene AT5G63090, which is expressed in a band of cells at the base of all lateral organs (Shuai *et al.*, 2002), encodes a protein that has been shown to interact directly with members of the bHLH family (Husbands *et al.*, 2007).

We interpret the reduced growth of the aerial part of *P35S::MtbHLH1-EAR* composite plants as an alteration of the nodule capacity to deliver products of symbiotic N fixation to the plant, as plant growth was found to be similar to WT in the presence of ammonium nitrate (without *S. meliloti* inoculation). The reduced growth could be caused by altered VBs or changes in the functioning of the cells where *MtbHLH1* is expressed, that is, VB pericycle cells and/or zone III UCs. It should be recalled that pericycle cells play a critical role for nutrient exchange with nearby tissues, and have been shown in different legume genera to exhibit an intense metabolic activity (Pate *et al.*, 1969), and that Abd-Alla *et al.* (2000) have reported that UCs from *Vicia faba* indeterminate nodules build up a symplasmic network through frequent plasmodesmata. Thus *c.* 30 times more plasmodesmata were counted between UCs, and between UCs and infected cells (ICs) than between ICs, suggesting a role for UCs in metabolite transport. Such a role has already been established in determinate nodules where UCs are specifically involved in synthesis and transport of ureides, the major product of N fixation transported in determinate nodules (Vance, 2002). In *Vicia faba* indeterminate nodules, uptake experiments with protoplasts isolated either from UCs or ICs, have led to the proposition that UCs are involved in bringing sugar from the phloem sap to the infected cells and transferring amino acids symbiotically produced by ICs to the peripheral vascular system (Peiter & Schubert, 2003; Peiter *et al.*, 2004). It is then tempting to propose that MtbHLH1 contributes to controlling nutrient exchange between nodule and root cells, that is, between ICs (source) and the rest of the plant (sink). Some of the genes, like the asparagine synthase or the ABC transporter genes, that showed reduced expression in *P35S::MtbHLH1-EAR* nodules may be involved in this process. Only a few differentially expressed genes were found in *P35S::MtbHLH1-EAR* nodules at 24 dpi compared with control nodules. Several of them were validated by independent qRT-PCR experiments and could be direct or indirect target genes of the MtbHLH1 TF. MtbHLH1 is likely to be a G-box DNA binder, as indicated by the presence of key amino acids (H-E-R-R) in the basic region of the bHLH domain, found in 44% of 638 plant bHLH proteins (Carretero-Paulet *et al.*, 2010). The MtbHLH1 protein, once dimerized, could physically bind to the G-box found in the promoters of some of the putative target genes revealed by transcriptome analyses, such as the asparagine synthase and a late nodulin genes, and directly induce their transcription. In the bean legume, a bHLH protein carrying H-E-R-R amino acids in the basic region has been shown to bind to the G-box motif of a gene encoding a seed-storage protein, the β-phaseolin (Kawagoe & Murai, 1996).

As far as early symbiotic stages are concerned, Complainville *et al.* (2003) have shown that *M. truncatula* nodule initiation induces symplasmic continuity between the root phloem and nodule initials such as the pericycle cells and immature sieve elements that will give rise to vascularization. The symplasmic field created precedes nodule cell division and allows the transport of macromolecules between root phloem and nodule (phloem unloading). MtbHLH1 might be involved in contributing to such cell to cell communications during early symbiotic stages, which could also explain the delay in nodulation observed in *P35S::MtbHLH1:EAR* roots. It would be very interesting in the future to analyse more specifically the transcriptome of nodule primordia cells and nodule UCs, for example by taking advantage of laser microdissection, to avoid dilution problems and thereby have a better understanding of their role and the consequences of MtbHLH1 alteration.

## Supporting Information

The following materials can be found in the online version of this article.

**Fig. S1** Alignment of the predicted MtbHLH1 bHLH domain with consensus sequences from plant bHLH proteins and protein sequence alignment between MtbHLH1 and its closest homologue from *Arabidopsis thaliana*, AT1G72210.

**Fig. S2** Box plot representations of noninoculated root architecture, following *Agrobacterium rhizogenes* transformation with an empty vector or a *P35S::MtbHLH1-EAR* construct.

**Table S1** Primers used for quantitative reverse-transcription polymerase chain reaction amplification of some MtbHLH1 candidate target genes

**Table S2** Putative target genes of the MtbHLH1 Transcription Factor obtained by transcriptome analyses

## References

[b1] Abd-Alla MH, Koyro H-W, Yan F, Schubert S, Peiter E (2000). Functional structure of the indeterminate *Vicia faba* L. root nodule: implications for metabolite transport. Journal of Plant Physiology.

[b2] Atchley WR, Terhalle W, Dress A (1999). Positional dependence, cliques, and predictive motifs in the bHLH protein domain. Journal of Molecular Evolution.

[b3] Auriac MC, Timmers AC (2007). Nodulation studies in the model legume *Medicago truncatula*: advantages of using the constitutive EF1alpha promoter and limitations in detecting fluorescent reporter proteins in nodule tissues. Molecular Plant–Microbe Interactions.

[b4] Benedito VA, Torres-Jerez I, Murray JD, Andriankaja A, Allen S, Kakar K, Wandrey M, Verdier J, Zuber H, Ott T (2008). A gene expression atlas of the model legume *Medicago truncatula*. Plant Journal.

[b5] de Billy F, Grosjean C, May S, Bennett M, Cullimore JV (2001). Expression studies on AUX1-like genes in *Medicago truncatula* suggest that auxin is required at two steps in early nodule development. Molecular Plant–Microbe Interactions.

[b6] Boisson-Dernier A, Chabaud M, Garcia F, Becard G, Rosenberg C, Barker DG (2001). *Agrobacterium rhizogenes*-transformed roots of *Medicago truncatula* for the study of nitrogen-fixing and endomycorrhizal symbiotic associations. Molecular Plant–Microbe Interactions.

[b7] Boualem A, Laporte P, Jovanovic M, Laffont C, Plet J, Combier JP, Niebel A, Crespi M, Frugier F (2008). MicroRNA166 controls root and nodule development in *Medicago truncatula*. Plant Journal.

[b8] Bougourd S, Marrison J, Haseloff J (2000). An aniline blue staining procedure for confocal microscopy and 3D imaging of normal and perturbed cellular phenotypes in mature Arabidopsis embryos. Plant Journal.

[b9] Brownlie P, Ceska T, Lamers M, Romier C, Stier G, Teo H, Suck D (1997). The crystal structure of an intact human Max-DNA complex: new insights into mechanisms of transcriptional control. Structure.

[b10] Carretero-Paulet L, Galstyan A, Roig-Villanova I, Martinez-Garcia JF, Bilbao-Castro JR, Robertson DL (2010). Genome-wide classification and evolutionary analysis of the bHLH family of transcription factors in Arabidopsis, poplar, rice, moss, and algae. Plant Physiology.

[b11] Combier JP, Frugier F, de Billy F, Boualem A, El-Yahyaoui F, Moreau S, Vernie T, Ott T, Gamas P, Crespi M (2006). MtHAP2-1 is a key transcriptional regulator of symbiotic nodule development regulated by microRNA169 in *Medicago truncatula*. Genes and Development.

[b12] Combier JP, Vernie T, de Billy F, El Yahyaoui F, Mathis R, Gamas P (2007). The MtMMPL1 early nodulin is a novel member of the matrix metalloendoproteinase family with a role in *Medicago truncatula* infection by *Sinorhizobium meliloti*. Plant Physiology.

[b13] Complainville A, Brocard L, Roberts I, Dax E, Sever N, Sauer N, Kondorosi A, Wolf S, Oparka K, Crespi M (2003). Nodule initiation involves the creation of a new symplasmic field in specific root cells of *Medicago* species. Plant Cell.

[b14] Frugier F, Poirier S, Satiat-Jeunemaitre B, Kondorosi A, Crespi M (2000). A Kruppel-like zinc finger protein is involved in nitrogen-fixing root nodule organogenesis. Genes and Development.

[b15] Gallavotti A, Zhao Q, Kyozuka J, Meeley RB, Ritter MK, Doebley JF, Pe ME, Schmidt RJ (2004). The role of barren stalk1 in the architecture of maize. Nature.

[b16] Gamas P, de Carvalho-Niebel F, Lescure N, Cullimore J (1996). Use of a subtractive hybridization approach to identify new *Medicago truncatula* genes induced during root nodule development. Molecular Plant–Microbe Interactions.

[b17] Godiard L, Niebel A, Micheli F, Gouzy J, Ott T, Gamas P (2007). Identification of new potential regulators of the *Medicago truncatula–Sinorhizobium meliloti* symbiosis using a large-scale suppression subtractive hybridization approach. Molecular Plant–Microbe Interactions.

[b18] Heim MA, Jakoby M, Werber M, Martin C, Weisshaar B, Bailey PC (2003). The basic helix–loop–helix transcription factor family in plants: a genome-wide study of protein structure and functional diversity. Molecular Biology and Evolution.

[b19] Hiratsu K, Matsui K, Koyama T, Ohme-Takagi M (2003). Dominant repression of target genes by chimeric repressors that include the EAR motif, a repression domain, in Arabidopsis. Plant Journal.

[b20] Husbands A, Bell EM, Shuai B, Smith HM, Springer PS (2007). LATERAL ORGAN BOUNDARIES defines a new family of DNA-binding transcription factors and can interact with specific bHLH proteins. Nucleic Acids Research.

[b21] Imaizumi-Anraku H, Kouchi H, Syono K, Akao S, Kawaguchi M (2000). Analysis of ENOD40 expression in alb1, a symbiotic mutant of *Lotus japonicus* that forms empty nodules with incompletely developed nodule vascular bundles. Molecular and General Genetics.

[b22] Kaiser BN, Finnegan PM, Tyerman SD, Whitehead LF, Bergersen FJ, Day DA, Udvardi MK (1998). Characterization of an ammonium transport protein from the peribacteroid membrane of soybean nodules. Science.

[b23] Karas B, Amyot L, Johansen C, Sato S, Tabata S, Kawaguchi M, Szczyglowski K (2009). Conservation of lotus and Arabidopsis basic helix–loop–helix proteins reveals new players in root hair development. Plant Physiology.

[b24] Kawagoe Y, Murai N (1996). A novel basic region/helix–loop–helix protein binds to a G-box motif CACGTG of the bean seed storage protein β-phaseolin gene. Plant Science.

[b25] Kevei Z, Vinardell JM, Kiss GB, Kondorosi A, Kondorosi E (2002). Glycine-rich proteins encoded by a nodule-specific gene family are implicated in different stages of symbiotic nodule development in *Medicago* spp. Molecular Plant–Microbe Interactions.

[b26] Koyama T, Furutani M, Tasaka M, Ohme-Takagi M (2007). TCP transcription factors control the morphology of shoot lateral organs via negative regulation of the expression of boundary-specific genes in Arabidopsis. Plant Cell.

[b27] Kuppusamy KT, Ivashuta S, Bucciarelli B, Vance CP, Gantt JS, Vandenbosch KA (2009). Knockdown of CELL DIVISION CYCLE16 reveals an inverse relationship between lateral root and nodule numbers and a link to auxin in *Medicago truncatula*. Plant Physiology.

[b28] Kuster H, Becker A, Firnhaber C, Hohnjec N, Manthey K, Perlick AM, Bekel T, Dondrup M, Henckel K, Goesmann A (2007). Development of bioinformatic tools to support EST-sequencing, *in silico*- and microarray-based transcriptome profiling in mycorrhizal symbioses. Phytochemistry.

[b29] Li X, Duan X, Jiang H, Sun Y, Tang Y, Yuan Z, Guo J, Liang W, Chen L, Yin J (2006). Genome-wide analysis of basic/helix–loop–helix transcription factor family in rice and Arabidopsis. Plant Physiology.

[b30] Libault M, Joshi T, Benedito VA, Xu D, Udvardi MK, Stacey G (2009). Legume transcription factor genes: what makes legumes so special?. Plant Physiology.

[b31] Limpens E, Ramos J, Franken C, Raz V, Compaan B, Franssen H, Bisseling T, Geurts R (2004). RNA interference in *Agrobacterium rhizogenes*-transformed roots of Arabidopsis and *Medicago truncatula*. Journal of Experimental Botany.

[b32] Lin WC, Shuai B, Springer PS (2003). The Arabidopsis LATERAL ORGAN BOUNDARIES-domain gene ASYMMETRIC LEAVES2 functions in the repression of KNOX gene expression and in adaxial–abaxial patterning. Plant Cell.

[b33] Lohar DP, Sharopova N, Endre G, Penuela S, Samac D, Town C, Silverstein KA, VandenBosch KA (2006). Transcript analysis of early nodulation events in *Medicago truncatula*. Plant Physiology.

[b34] MacAlister CA, Ohashi-Ito K, Bergmann DC (2007). Transcription factor control of asymmetric cell divisions that establish the stomatal lineage. Nature.

[b35] Marini AM, Springael JY, Frommer WB, Andre B (2000). Cross-talk between ammonium transporters in yeast and interference by the soybean SAT1 protein. Molecular Microbiology.

[b36] Massari ME, Murre C (2000). Helix–loop–helix proteins: regulators of transcription in eukaryotic organisms. Molecular and Cellular Biology.

[b37] Mathesius U (2008). Auxin: at the root of nodule development?. Functional Plant Biology.

[b38] Matsui K, Hiratsu K, Koyama T, Tanaka H, Ohme-Takagi M (2005). A chimeric AtMYB23 repressor induces hairy roots, elongation of leaves and stems, and inhibition of the deposition of mucilage on seed coats in Arabidopsis. Plant and Cell Physiology.

[b39] Mergaert P, Nikovics K, Kelemen Z, Maunoury N, Vaubert D, Kondorosi A, Kondorosi E (2003). A novel family in *Medicago truncatula* consisting of more than 300 nodule-specific genes coding for small, secreted polypeptides with conserved cysteine motifs. Plant Physiology.

[b40] Mitsuda N, Iwase A, Yamamoto H, Yoshida M, Seki M, Shinozaki K, Ohme-Takagi M (2007). NAC transcription factors, NST1 and NST3, are key regulators of the formation of secondary walls in woody tissues of Arabidopsis. Plant Cell.

[b41] Moreau S, Verdenaud M, Ott T, Letort S, de Billy F, Niebel A, Gouzy J, de Carvalho-Niebel F, Gamas P (2011). Transcription reprogramming during root nodule development in *Medicago truncatula*. PLoS ONE.

[b42] van Noorden GE, Kerim T, Goffard N, Wiblin R, Pellerone FI, Rolfe BG, Mathesius U (2007). Overlap of proteome changes in *Medicago truncatula* in response to auxin and *Sinorhizobium meliloti*. Plant Physiology.

[b43] Ohashi-Ito K, Bergmann DC (2006). Arabidopsis FAMA controls the final proliferation/differentiation switch during stomatal development. Plant Cell.

[b44] Ohashi-Ito K, Bergmann DC (2007). Regulation of the Arabidopsis root vascular initial population by LONESOME HIGHWAY. Development.

[b45] Oldroyd GE, Downie JA (2008). Coordinating nodule morphogenesis with rhizobial infection in legumes. Annual Review of Plant Biology.

[b46] Parizot B, Laplaze L, Ricaud L, Boucheron-Dubuisson E, Bayle V, Bonke M, De Smet I, Poethig SR, Helariutta Y, Haseloff J (2008). Diarch symmetry of the vascular bundle in Arabidopsis root encompasses the pericycle and is reflected in distich lateral root initiation. Plant Physiology.

[b47] Pate JS, Gunning BES, Briarty LG (1969). Ultrastructure and functioning of the transport system of the leguminous root nodule. Planta.

[b48] Peiter E, Schubert S (2003). Sugar uptake and proton release by protoplasts from the infected zone of *Vicia faba* L. nodules: evidence against apoplastic sugar supply of infected cells. Journal of Experimental Botany.

[b49] Peiter E, Yan F, Schubert S (2004). Amino acid export from infected cells of *Vicia faba* root nodules: evidence for an apoplastic step in the infected zone. Physiologia Plantarum.

[b50] Peret B, De Rybel B, Casimiro I, Benkova E, Swarup R, Laplaze L, Beeckman T, Bennett MJ (2009). Arabidopsis lateral root development: an emerging story. Trends in Plant Science.

[b51] Ramsay NA, Glover BJ (2005). MYB-bHLH-WD40 protein complex and the evolution of cellular diversity. Trends in Plant Science.

[b52] Rolland-Lagan AG (2008). Vein patterning in growing leaves: axes and polarities. Current Opinion in Genetics and Development.

[b53] Schubert S, Sattelmacher B, Horst WJ (2007). The apoplast of indeterminate legume nodules: compartment for transport of amino acids, amides and sugars?. The apoplast of higher plants: compartment of storage, transport and reactions.

[b54] Semiarti E, Ueno Y, Tsukaya H, Iwakawa H, Machida C, Machida Y (2001). The ASYMMETRIC LEAVES2 gene of *Arabidopsis thaliana* regulates formation of a symmetric lamina, establishment of venation and repression of meristem-related homeobox genes in leaves. Development.

[b55] Shi L, Twary SN, Yoshioka H, Gregerson RG, Miller SS, Samac DA, Gantt JS, Unkefer PJ, Vance CP (1997). Nitrogen assimilation in alfalfa: isolation and characterization of an asparagine synthetase gene showing enhanced expression in root nodules and dark-adapted leaves. Plant Cell.

[b56] Shuai B, Reynaga-Pena CG, Springer PS (2002). The lateral organ boundaries gene defines a novel, plant-specific gene family. Plant Physiology.

[b57] Sprent JI, James EK (2007). Legume evolution: where do nodules and mycorrhizas fit in?. Plant Physiology.

[b58] Stevens JD, Roalson EH, Skinner MK (2008). Phylogenetic and expression analysis of the basic helix–loop–helix transcription factor gene family: genomic approach to cellular differentiation. Differentiation.

[b59] Stougaard J (2000). Regulators and regulation of legume root nodule development. Plant Physiology.

[b60] Takeda T, Suwa Y, Suzuki M, Kitano H, Ueguchi-Tanaka M, Ashikari M, Matsuoka M, Ueguchi C (2003). The OsTB1 gene negatively regulates lateral branching in rice. Plant Journal.

[b61] Timmers AC, Auriac MC, Truchet G (1999). Refined analysis of early symbiotic steps of the *Rhizobium*–*Medicago* interaction in relationship with microtubular cytoskeleton rearrangements. Development.

[b62] Toledo-Ortiz G, Huq E, Quail PH (2003). The Arabidopsis basic/helix–loop–helix transcription factor family. Plant Cell.

[b63] Vance CP, Waisel Y, Eshel A, Kafkati U (2002). Root–bacteria interactions: symbiotic nitrogen fixation. Plant roots: the hidden half, 3rd edn.

[b64] Vasse J, De Billy F, Camut S, Truchet G (1990). Correlation between ultrastructural differentiation of bacteroids and nitrogen fixation in alfalfa nodules. Journal of Bacteriology.

[b65] Vernié T, Moreau S, de Billy F, Plet J, Combier JP, Rogers C, Oldroyd G, Frugier F, Niebel A, Gamas P (2008). EFD Is an ERF transcription factor involved in the control of nodule number and differentiation in *Medicago truncatula*. Plant Cell.

[b66] Yang WC, de Blank C, Meskiene I, Hirt H, Bakker J, van Kammen A, Franssen H, Bisseling T (1994). *Rhizobium* nod factors reactivate the cell cycle during infection and nodule primordium formation, but the cycle is only completed in primordium formation. Plant Cell.

[b67] Yano K, Tansengco ML, Hio T, Higashi K, Murooka Y, Imaizumi-Anraku H, Kawaguchi M, Hayashi M (2006). New nodulation mutants responsible for infection thread development in *Lotus japonicus*. Molecular Plant–Microbe Interactions.

[b68] Zgurski JM, Sharma R, Bolokoski DA, Schultz EA (2005). Asymmetric auxin response precedes asymmetric growth and differentiation of asymmetric leaf1 and asymmetric leaf2 Arabidopsis leaves. Plant Cell.

