# Supplementary figures and images for: MtbHLH1, a bHLH transcription factor involved in Medicago truncatula nodule vascular patterning and nodule to plant metabolic exchanges
Source: New Phytol. 2011 Jul;191(2):391–404. doi: 10.1111/j.1469-8137.2011.03718.x (PMC3206218; doi:10.1111/j.1469-8137.2011.03718.x)

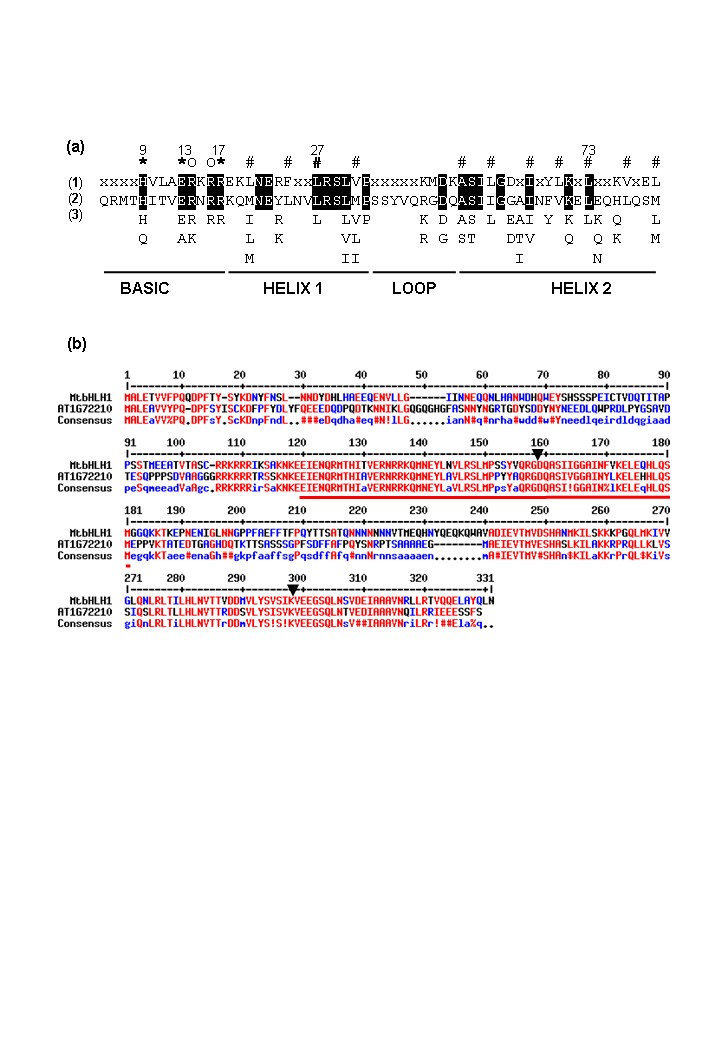

Supplement: Supplementary file 1 [file nph0191-0391-SD1.tif]
