# Supplementary material for: MtbHLH1, a bHLH transcription factor involved in Medicago truncatula nodule vascular patterning and nodule to plant metabolic exchanges
Source: New Phytol. 2011 Jul;191(2):391–404. doi: 10.1111/j.1469-8137.2011.03718.x (PMC3206218; doi:10.1111/j.1469-8137.2011.03718.x)
Supplement: Supplementary file 2 [file nph0191-0391-SD2.doc]

**Supporting Information Figs S1-S2**

**Fig. S1** (a) Alignment of the predicted MtbHLH1 bHLH domain with consensus sequences from plant bHLH proteins

(1) bHLH domain consensus (Heim *et al.*, 2003)

(2) Predicted MtbHLH1 bHLH domain

(3) Conserved amino acid positions observed in more than 50% of the plant bHLH sequences with an occurrence of more than 10% (Carretero-Paulet *et al.*, 2010)

Amino acids that have been predicted to be important for contact with a nucleotide base (*), DNA backbone (circles o) and protein-protein interactions (#) are indicated, as in (Heim *et al.*, 2003). Amino acid numbers are from Carretero-Paulet *et al.*, (2010).

(b) Protein sequence alignment (using Multalin software, Corpet, 1988) between MtbHLH1 and its closest homologue from *A.thaliana*, AT1G72210

The bHLH motif is underlined in red. Intron positions present in the genes of both proteins are indicated

by black arrows.

**Fig. S2** Box plot representations of non inoculated root architecture, following *A. rhizogenes* transformation with an empty vector (*n* = 50) (labelled ‘control’) or a 35S:MtbHLH1:EAR construct (*n* = 56).

(a) total number of root tips per plant.

(b) length of the longest root (in cm).

Plants were grown in 1 mM ammonium nitrate. Transformed roots were selected by kanamycin resistance and systematically verified with a DsRed marker present in the T-DNA construct.

**References**

**Carretero-Paulet L, Galstyan A, Roig-Villanova I, Martinez-Garcia JF, Bilbao-Castro JR, Robertson DL. 2010.** Genome-wide classification and evolutionary analysis of the bHLH family of transcription factors in Arabidopsis, poplar, rice, moss, and algae. *Plant Physiol* **153**: 1398-1412.

**Corpet F. 1988.** Multiple sequence alignment with hierarchical clustering. *Nucleic Acids Res* **16**: 10881-10890.

**Heim MA, Jakoby M, Werber M, Martin C, Weisshaar B, Bailey PC. 2003.** The basic helix-loop-helix transcription factor family in plants: a genome-wide study of protein structure and functional diversity. *Mol Biol Evol* **20**: 735-747.
